# The effects of health expenditure on infant mortality in sub-Saharan Africa: evidence from panel data analysis

**DOI:** 10.1186/s13561-020-00262-3

**Published:** 2020-03-06

**Authors:** Girmay Tsegay Kiross, Catherine Chojenta, Daniel Barker, Deborah Loxton

**Affiliations:** 1 Department of Public Health, College of Health Sciences, Debre Markos University, Debre Markos, Ethiopia; 2 Research Centre for Generational Health and Ageing, Faculty of Health and Medicine, University of Newcastle, Newcastle, New South Wales Australia; 3 School of Medicine and Public Health, Faculty of Health and Medicine, University of Newcastle, Newcastle, New South Wales Australia

**Keywords:** Health care expenditure, Infant mortality, Random effects

## Abstract

**Introduction:**

Although health expenditure in sub-Saharan African countries is the lowest compared with other regions in the world, most African countries have improved their budget allocations to health care over the past 15 years. The majority of health care sources in sub-Saharan Africa are private and largely involve out-of-pocket expenditure, which may prevent healthcare access. Access to healthcare is a known predictor of infant mortality. Therefore the objective of this study is to determine the impact of health care expenditure on infant mortality in sub-Saharan Africa.

**Methods:**

The study used panel data from World Bank Development Indictors (WDI) from 2000 to 2015 covering 46 countries in sub-Saharan Africa. The random effects model was selected over the fixed effects model based on the Hausman test to assess the effect of health care expenditure on infant and neonatal mortality.

**Results:**

Both public and external health care spending showed a significant negative association with infant and neonatal mortality. However, private health expenditure was not significantly associated with either infant or neonatal mortality. In this study, private expenditure includes funds from households, corporations and non-profit organizations. Public expenditure include domestic revenue as internal transfers and grants, transfers, subsidies to voluntary health insurance beneficiaries, non-profit institutions serving households or enterprise financing schemes as well as compulsory prepayment and social health insurance contributions. External health expenditure is composed of direct foreign transfers and foreign transfers distributed by government encompassing all financial inflows into the national health system from outside the country.

**Conclusion:**

Health care expenditure remains a crucial component of reducing infant and neonatal mortality in sub-Saharan African countries. In the region, where health infrastructure is largely underdeveloped, increasing health expenditure will contribute to progress towards reducing infant and neonatal mortality during the Sustainable Development Goals (SDGs) era. Therefore, governments in the region need to increase amounts allocated to health care service delivery in order to reduce infant mortality.

## Introduction

The risk of a child dying before the age of one was highest in the World Health Organization African Region (51 per 1000 live births), which is over six times higher than that in the WHO European Region (8 per 1000 live births) [1]. Although sub-Saharan African (SSA) countries have achieved remarkable improvement in infant survival rates since the introduction of the Millennium Development Goals (MDGs), infant mortality in SSA continues to be the highest among all global regions [1]. Different socioeconomic factors are considered responsible for the high rate of infant mortality in developing countries; the most commonly sighted factors in previous studies have been the level of female education, per capita income, general environmental cleanliness and expenditure on health, [2].

Among the SDGs, which are targets that all countries agreed to try to achieve by 2030, is the goal to “ensure healthy lives and promote wellbeing for all at all ages” [3]. More specifically, it focuses on a substantial reduction in the global child mortality rate as well as “substantially increase health financing” [4]. Public expenditure on health represents one of the key drivers in meeting these important elements of the SDGs [3]. Health expenditure is fundamental to the ability of health systems to maintain and improve human welfare; without financing, skilled and appropriate health workers would not be employed, medical equipment would not be available and health promotion or prevention of disease would not take place [5]. Health expenditure reflects the overall level of consumption of health goods? and services by the population across countries [6]. Investing in the health care system will not only lead to healthier lives, it also creates employment, enhances political and social stability, and contributes to economic growth and productivity [6].

In 2015, the world spent USD 7.3 trillion on health, which is close to 10% of global gross domestic product (GDP) [7]. Health expenditure per share of GDP was greatest in high-income countries at nearly 12% on average [7]. In low-income countries, health expenditure accounts on average for 7% of GDP, and in middle-income countries 6% [7, 8]. Globally the health sector has consistently grown faster than economic growth over the past 15 years [7, 8]. Between 2000 and 2015, the global health economy grew in real terms at an average annual rate of 4.0% compared with 2.8% for the global economy [6]. The health economy in low-income and lower middle-income countries has grown even faster, at more than 6.0% on average [6]. In 2015, the average share of external resources to health spending in the 31 low-income countries was around 30% while in the 50 lower-middle and 57 upper-middle countries it was only 3% and less than 1%, respectively [6, 9].

The way health care is financed varies considerably across countries [10]. Middle-income and high-income countries tend to have a higher share of health spending that is funded from compulsory prepaid sources, such as government budgets (from various types of taxes) and social health insurance contributions [10, 11]. Public funding has increased slightly over the past 15 years from an average of 48% to 51% of current health spending in middle-income countries and from 66% to 70% in high-income countries [12]. In low-income countries domestic government sources have declined from 30% to 22% as aid increased from 20% to 30% [9].

According to the Abuja Declaration in 2001, the African Union member heads of state agreed to allocate at least 15% of annual expenditure to health care [13]. Fifteen years later in 2014, most African countries had increased the proportion of total public expenditure allocated to health care [14].. Although, health expenditure in SSA is the lowest compared with other regions, most African countries have improved their budget allocations to health over the past 15 years [15]. The source of health care financing in the SSA region was mostly from private sources and largely out-of-pocket (OOP) expenditure; WHO estimates that up to 10% of the population in the region suffer a financial catastrophe each year due to out of pocket OOP expenditure, with up to 4 % pushed under the poverty line of the region [16]. Government health expenditure in Africa does not always go up with increasing national income or government revenues. For instance, high-income countries in Africa did not systemically allocate higher priority to health care in their government spending [14]. In contrast, a few lower income countries have allocated more than 15% of their public spending to the sector (Ethiopia, Gambia, and Malawi) [14].

The level of health expenditure in a nation is one important measure of the level of health investment; thus, it is recognized as an important input, just like exercising and dieting, in improving health outcomes [17]. Previous studies have reported a positive effect of health expenditure on health outcomes such as infant and child mortality [18, 19], whereas other studies have reported that health expenditure was not a crucial determinant of health outcomes [20]. For instance, a study conducted in 2017 among SSA countries reported that public health expenditure has a significant influence in reducing infant mortality, whereas private health expenditure is not significantly associated with infant mortality [19]. Other studies have suggested that the effect of public health expenditure is stronger than that of private health expenditure [21]. Similar findings from a panel of Indian states indicated that public expenditure on healthcare reduces infant mortality [22]. Another study conducted on health spending and infant mortality among Asian countries found that low-income countries which allocate a reasonable proportion of expenditure on health enjoy relatively lower infant mortality [23]. On the other hand, studies conducted among 15 south Asian countries showed that total health expenditure, public health expenditure and private health expenditure has a significant effect in reducing infant mortality rate and the extent of the effect is greater with private health expenditure than public health expenditure [24]. Funding form the external sources, such as bilateral and multilateral agencies, to increase their financial support to the health systems in low-income and high-disease-burden countries have an important role for improving the population health status like reducing infant mortality [25]. Special assistance from bilateral and multilateral agencies to recovering from war or dealing with severe hunger may also improve the health status of a nation in low and middle income countries [25]. Different studies in African countries has been shown that health expenditure from external source was associated with reduced prevalence and severity of diarrhea [26]. It was reducing the prevalence of malaria as well as improved quality of self-reported health and it was also reduced the overall disease severity [27, 28].

Based on the previous studies, there are no clear-cut conclusions to be drawn regarding the impact of health expenditure on infant mortality. Furthermore, the impact of external health expenditure was not addressed in the previous studies. In addition, previous studies have mostly failed to control for other determinants of infant mortality, such as social determinants, prevalence of diseases, environmental conditions, the use of preventive health care/immunization and population dynamics. This may have the potential to lead to a bias in the results obtained in such studies. For instance, it is known that immunization against some known diseases over the years has contributed to a decline in infant mortality rates and the high incidence of HIV/AIDS has caused a significant increase in mortality rates. Failure to account for these factors may lead to misleading results. Most of the previous studies classified the source of health expenditure as public and private. However, in the new WHO report the source of health expenditure is classified as public, private and external.

While most of the previous studies focused on the impact of public and private expenditures only, in this study, the impact of health expenditure and infant mortality was explained in terms of public, private and external expenditures separately. Therefore, this study may give new insights into the impact of health expenditure. In addition, the WHO report summarizes the latest internationally comparable data on health spending in all WHO member states between 2000 and 2015 [6, 29]. The report also uses the new international classification for health expenditures in the revised System of Health Accounts [6, 29]. These classifications enable the presentation of detailed information on the role of governments, households and donors in funding health services and the financing arrangements through which these funds are channelled and spent [29]. It also helps to obtain a clear and transparent picture of health financing systems, including information that is relevant to health policy about the structure and flows of funds (transactions). This includes indicators comparable across countries and over time that can contribute to the assessment of the performance of health financing systems [30]. This new version of WHO’s Global Health Expenditure Database (GHED) includes new estimates of health expenditures in 2015, revised data series for each country and each year from 2000 to 2015 as well as new classifications. This database improves the comparability and policy-relevance of the estimates [11, 29]. In addition, it improves the data quality, which can produce sound results for policy makers and decision makers. This study may update scholars since this study used current data. Therefore, the aim of this study is to assess the impact of health expenditure on infant mortality among SSA countries.

## Method

### Data and variables

The study used pooled panel data from 2000 to 2015 for 46 countries in SSA. The source of data for this study was the World Bank Development Indictors (WDI) [31]. We used infant mortality rate and neonatal mortality rate as outcome variables. The infant mortality rate is measured as the death of a child less than 1 year old per 1000 live births and the neonatal mortality rate is measured as the death of a child less than 28 days per 1000 live births.

Predictor variables included total health expenditure measured as percentage of GDP and income per head as measured by GDP per capita (Additional file 1). Higher health care expenditure is expected to be associated with lower infant and neonatal mortality. Different population age groups, namely those under 14 years and above 65 years, were measured as a percentage of the total population. These were included to control for different country demographic structures. Relative to the younger population, the population age group above 65 years is expected to increase infant mortality outcomes by increasing death rates. To control for the varying levels of infant and neonatal mortality in SSA, HIV prevalence rate, maternal mortality ratio, fertility rate, access to improved water and sanitation, measles vaccination coverage, and school enrolment were included in the model. In addition, we used immunization rate as a proxy to measure the effect of the use of preventive health care services on health outcomes such as infant mortality.

### Model

To model the two health status outcomes (infant mortality and neonatal mortality), we used random effects models on the pooled panel data from 2000 to 2015 for 46 countries in SSA [21]. To predict health status using these models, we added the covariates total health expenditure as a percentage of real national income, gross domestic product per capita real income, which acts as a control variable for the demand for health services and other economic factors. The total health expenditure is further grouped in to public health expenditure, private health expenditure and external health expenditure. The demographic variables represent population age groups of under 14 and over 65 years age, respectively, and expressed as a percentage of total population. In this model, a random effect was added for country to control for unobserved heterogeneity and the outcome measure and predictors were transformed using a logarithmic function where appropriate. In addition we used demographic variables to control variation across the countries The modelling approach for the panel data from previous studies is as follows [21].

$$ {\mathrm{Y}}_{\mathrm{i}\mathrm{t}}={\mathbf{X}}_{\mathbf{it}}\boldsymbol{\upbeta} +{\upupsilon}_{\mathrm{i}}+{\upvarepsilon}_{\mathrm{i}\mathrm{t}} $$

Where Y_it_ is the outcome variable in country i at time t, **X** is the matrix of predictor variables, including the intercept, and **β** is the matrix of fixed regression coefficients. The total variation in the model is broken up into two parts. Between country error represented by the random effect term υ_i_ and within country error denoted by ε_it_.

In most panel data analysis, there was the need to test for random effects or panel effects in the model. The Breuch-Pagan Lagrange Multiplier (LM) test was used to make a decision between random effects regression and simple OLS regression. The null hypothesis in the LM test is that variances across the countries is zero. This is, no significant difference across units (i.e. no panel effect). Here we rejected the null hypothesis and conclude that random effects are appropriate at the 5% level of confidence (*p*-value < 0.001; 95% CI) [32]. Secondly, the Hausman’s specification test was employed to compare estimates from the random effects and the fixed effects models. The null hypothesis in the Hausman’s test is that the error term for country is not correlated with the predictors. Here we failed to reject the null hypothesis and conclude that random effects is appropriate at the 5% level of confidence (*P*-value = 0.077) [32].

## Results

The majority of the population in SSA countries (55.37%) were aged 15–64 years. Of the remaining population, 41.1% were aged less than 14 years and 3.53% were above 65 years.

Total health expenditure is the sum of public, private and external expenditure [8]. It covers the provision of health services (preventive and curative), family planning activities, nutrition activities, and emergency aid designated for health [8]. In this study, the average health expenditure per capita was around US$209. The average health expenditure as percentage of GDP was approximately 6%. Of this, private expenditure was 48% of current health expenditures, which was funded from domestic private sources. Domestic private sources include funds from households, corporations and non-profit organizations. Of the total health expenditure, 35% was funded from domestic public sources, which includes domestic revenue as internal transfers and grants, transfers, subsidies to voluntary health insurance beneficiaries, non-profit institutions serving households or enterprise financing schemes as well as compulsory prepayment and social health insurance contributions.

The remaining 18% was from external sources, composing of direct foreign transfers and foreign transfers distributed by government encompassing all financial inflows into the national health system from outside the country. External sources include either flow through the government scheme or were channelled through non-governmental organizations or other schemes.

The out-of-pocket payment for health care SSA was also one of the highest compared with other regions as 47% of the total health expenditure in this region was covered by out-of-pocket expenditure (Table 1).

**Table 1:** Descriptive statistics of the effect of health expenditure on infant mortality in sub-Saharan African countries

Variable	Mean	Std dev	Min	Max
Health expenditure per capita	209.04	824.49	4.43	8148.80
Health expenditure per GDP	5.53	2.27	.84	19.723
Domestic private expenditure	48.35	19.02	2.54	97.38
Domestic public expenditure	34.21	18.75	2.62	96.99
External expenditure	18.03	16.51	0	85.06
Infant mortality rate	61.39	26.49	11.4	142
Neonatal mortality rate	30.43	10.75	7	57.2
Fertility rate	4.8	1.39	1.36	7.68
Maternal mortality ratio	547.69	347.52	9	2650
HIV prevalence rate	5.04	6.61	0.1	28.4
Measles vaccination coverage	74.39	18.70	16	99
Population aged under 14 years	41.1	6.46	19.40	50.22
Population aged 15–64 years	55.37	5.39	47.24	70.78
Population aged above 65 years	3.53	1.24	2.17	9.95
Access to improved water	59.85	20.17	8.8	99.8
Access to improved sanitation	29.75	26.80	2.4	98.4

### Health expenditure and infant mortality

The results from the random effects model show that increased health expenditure reduced infant mortality at 5% level of significance. An increase in total health expenditure per capita was negatively associated with infant mortality. A 1% increase in total health expenditure per capita was associated with a reduction in infant mortality of approximately 0.1% (CI: − 0.392, − 0.241; *p* < 0.0001). An increase in external health expenditure was significant in reducing infant mortality by approximately 0.03% (CI: − 0.046, − 0.014; *p* < 0.0001). Public health expenditure from domestic sources was also significantly associated with reduced infant mortality. A 1% increase in public health expenditure per capita was associated with a reduction in infant mortality of approximately 0.025% (CI: − 0.048, − 0.002; *p* = 0.034). Real GDP per capita was significantly associated with infant mortality; an increase in GDP per capita was negatively associated with infant mortality. An increase in GDP per capita was significantly associated with a reduction in infant mortality at 5% significance level. A 1% increase in real GDP per capita led to an improvement of infant mortality of approximately 0.2% (− 0.392, − 0.241, *p* = 0.001; Table 2).

**Table 2:** The effect of health expenditure on infant mortality in sub-Saharan African countries

Variable	Random effectss models			
	Model 1		Model 2	
	Estimate	95% CI	Estimate	95% CI
ln RGDPpc	−0.210	− 0.275, − 0.145*	− 0.317	− 0.392, − 0.241*
lnTHE	− 0.116	− 0.141, − 0.092*		
LnPuHE			− 0.025	− 0.048, − 0.002*
InPrHE			− 0.016	− 0.051, 0.018
LnExtHE			− 0.030	− 0.046, − 0.014*
lnfertility rate	0.263	0.092, 0.435*	0.372	0.188, 0.556*
lnHIV	0.081	0.047, 0 .115*	0.098	0.062, 0.136*
lnMMR	0.090	0.047, 0.133*	0.110	0.065, 0.155*
lnMeze	−0.003	−0.058, 0.066	− 0.063	− 0.132, 0.004
Lnsanitation (S)	− 0.013	− 0.041, 0.068	0.001	− 0.057, 0.059
lnUrbanizatio(U)	0.082	−0.047, 0.212	0.045	−0.093, 0.184
lnimproved water	−0.327	−0.442, − 0.212*	−0.195	− 0.320, − 0.069*
Lnpop	0.213	0.063, 0 .365*	0.277	0.114, 0.442*
lnEduc	−0.100	−0.157, − 0.039*	−0.073	− 0.140, − 0.006*
R-squared	0.832		0.817	
Observations	545		545	

Note: *significant at 5%; (1) is model with aggregate health care expenditure; and (2) is model with total health expenditure decomposed into public, private and external health expenditure

### Health expenditure and neonatal mortality

The results from the random effects models show that increasing health expenditure reduce neonatal mortality at 5% level of significance. An increase in health expenditure was negatively associated with neonatal mortality. A 1% increase in total health expenditure per capita reduced neonatal mortality by approximately 0.1% (CI: − 0.108, − 0.069; *p* < 0.0001). In this study, public health expenditure was negatively associated with neonatal mortality. A 1% increase in public expenditure per capita was negatively associated with a reduction in neonatal mortality by approximately 0.04%? (CI: − 0.055, − 0.021; *p* < 0.0001). Both private and external expenditure were not significantly associated with a reduction in neonatal mortality. The real GDP per capita was significantly associated with neonatal mortality; an increase in GDP per capita was negatively associated with neonatal mortality. A 1% increase in real GDP per capita led to an improvement in neonatal mortality of approximately 0.2% (CI: − 0.275, − 0.159; *p* = 0.001; Table 3).

**Table 3:** The effect of health expenditure on neonatal mortality in sub-Saharan African countries

Variable	Random effects models			
	Model 1		Model 2	
	Estimate	95% CI	Estimate	95% CI
LnGDP	−0.121	−0.173, − 0.070*	−0.217	− 0.275, − 0.159*
lnTHE	−0.088*	− 0.108, − 0.069*		
LnPuHE			−0.038	−0.055, − 0.021*
InPrHE			−0.017	−0.0431, 0.008
LnExtHE			−0.003	−0.015, 0 .008
lnHIV	0.031	−0.0003, 0.062	0.043	0.010, 0.077*
Ln MMR	0.103	0.068, 0.140*	0.146	0.109, 0.184*
Lnsanitation	−0.012	−0.056, 0.032	−0.025	− 0.072, 0.022
lnUR	0.080	−0.036, 0.193	0.013	−0.133, 0.107
lnaccess water	−0.231	−0.318, − 0.143*	−0.164	− 0.259, − 0.069*
Lnage65+	0.251	0.130, 0.372*	0.285	0.158, 0.412*
lnEDU	−0.112	−0.156, − 0.068*	−0.125	− 0.174, − 0.075*
R-squared	0.832		0.792	
Observations	540		540	

Note: *significant at 5%; (1) is model with aggregate health care expenditure; and (2) is model with total health expenditure decomposed into public, private, and external sources

## Discussion

The objective of this study was to assess the impact of health expenditure on infant mortality among SSA countries. The findings from this study showed that the overall health expenditure per capita was significantly associated with infant and neonatal mortality. A 1% increase in health expenditure per capita, irrespective of the source, significantly reduced infant and neonatal mortality by approximately 0.1% in the random effect model. This finding is similar to previous studies conducted in Africa and Asia [21, 22]. During the same period from 2000 to 2015 the rate of infant mortality in Africa has been substantial decreased, especially in a country such as Botswana, Rwanda, and Ethiopia [33].

Health expenditure is fundamental to the ability of health systems to maintain and improve human welfare; without financing, skilled and appropriate health workers would not be employed, medical equipment would not be available and health promotion or prevention of disease would not take place [5]. Health expenditure reflects the overall level of consumption of health goods and services by the population across countries [6]. Investing in the health care system will not only lead to healthier lives, it also creates employment, enhances political and social stability, and contributes to economic growth and productivity [6].

In this study, both public and external health expenditure were significantly negatively associated with infant mortality. However, private health expenditure was not significantly associated with either neonatal or infant mortality. The finding from this study is similar to a previous study, which shows that public health expenditure and infant mortality have a negative relationship [18].

Several previous studies have reported a positive effect of health expenditure on health outcomes such as infant and child mortality [18, 19], whereas other studies have reported that health expenditure is not a crucial determinant of health outcomes [20]. For instance, a study conducted among SSA countries in 2017 reported that public health expenditure has a significant influence on reducing infant mortality, whereas private health expenditure is not significantly associated with infant mortality [19]. Other studies have suggested that the effect of public health expenditure is stronger than that of private health expenditure [21]. Similar findings from a panel of Indian states indicated that public expenditure on healthcare reduces infant mortality [22]. Another study conducted on health spending and infant mortality among Asian countries found that low-income countries which allocate a reasonable proportion of expenditure on health enjoy relatively lower infant mortality [23]. On the other hand, study conducted among 15 south Asian countries showed that total health expenditure, public health expenditure and private health expenditure have a significant effect on reducing the infant mortality rate and that the effect of private health expenditure is greater than that of public health expenditure [24]. Another study conducted among 34 Asian countries reported that private spending on health does not have a significant benefit on infant mortality, which is similar with the finding of this study. High public health expenditure in a nation may be used for providing and developing health facilities and improving health system operations [34].

In this study external health expenditure and infant mortality have a significant association; as the health expenditure from the external sources increased, the infant mortality was decreased. The finding from this study supports previous findings. For example, a micro-level study from Nigeria showed that, children born in areas that received an aid project had a lower mortality than children born in areas that did not received an aid project [35]. Another large scale study that used data from 135 countries showed that healthcare aid had a statistically significant and positive effect on the infant mortality rate, a doubling of aid led to an approximately 1.3% reduction in infant mortality rates [36]. Another study conducted by Mishra and Newhouse (2009) found that a doubling of health aid was associated with a 2% drop in infant mortality, implying that increasing per capita health aid by US$1.60 per year was associated with 1.5 fewer infant deaths per thousand births [37]. However one of the earlier study in this field reported that, aid had no significant effect on health indicators [38]. The reason for this controversy may be the data were pooled together nations with the different economy.

In SSA the main source of private expenditure is out-of-pocket, which may create a burden on citizens with low incomes and those with poor health states [39]. In addition, private out-of-pocket health expenditure is a household catastrophic and results in household financial hardship and may push millions of people into extreme poverty each year [39]. In general, low-income economies have a higher share of private health expenditure than do middle- and high-income countries, and out-of-pocket expenditure (direct payments by households to providers) makes up the largest proportion of private expenditure [40]. According to the a 2017 WHO report in, 800 million people spend more than 10% of their annual budget on health care, and 100 million people are pushed into extreme poverty each year due to this out-of-pocket health expenditure [6, 39].

The findings of this study suggest that increasing health expenditure remains an important step in reducing infant and neonatal mortality in SSA countries. Increasing health expenditure, especially public health expenditure, is used to improve health service quality and accessibility by providing and developing health facilities and improving health system operations. In addition, public spending on essential health services such as immunization, communicable diseases, preventive health services, and food safety is justified by disease reduction and furthermore it reduces mortality rates.

Findings from other studies also show that health expenditure is an important determinant of child health outcomes; health expenditure has a significant negative relationship with infant mortality [21] . In contrast, other studies show that the effect of health expenditure on infant mortality is either small or statistically insignificant [20, 41].

In SSA where health infrastructure is largely underdeveloped, increasing health expenditure will mean significant progress towards reducing infant and neonatal mortality during the SDGs era. While this current study provides evidence in support of increasing health care expenditure to reduce infant mortality, in Africa public health expenditure does not systematically prioritize primary care or more service accessibility for poor people. The rich benefit mostly from public health funds. In this case, misallocation and poor management of public health spending means population health and infant mortality may worsen even as health care expenditure increases [42].

In addition to the effect of health expenditure, urban population growth rate was significantly associated with infant mortality. As urbanization increased, the rate of infant mortality decreased. Urbanization was used as a measure of access to health services, particularly in the urban centres. In developing countries, most health services are clustered around the urban areas. Therefore, an increase in urbanization may increase access and quality of maternal and child health services. The finding from this study is opposite to the findings in a previous study conducted in SSA [19]. The result in the previous study showed that an increase in the urban population growth rate results in an increase in the infant mortality rate [19]. The author suggests that pressure on health facilities in urban areas, which is characteristic of many developing regions due to the high migration rates to urban centres, results in a rise in mortality rates [19].

This study also found evidence of access to improved water being significantly associated with infant mortality. The results indicated that a 1% increase in the proportion of the population with access to improved water led to a reduction in infant mortality by approximately 3%. In addition, primary school enrolment rate was significant in reducing infant and neonatal mortality. This result is supported by previous studies which argue that improvements in education will lead to improvement in health outcomes since people become efficient in the use of health care resources and take good care of their infant health by seeking appropriate health care [20, 43].

### Strengths and limitations of the study

A limitation of this study was a high rate of missing data for some variables, such as the ratio of physician to population, which resulted in these variables being excluded from the study. In particular, it is possible that not all of the World Bank indicators were reported for each year. The World Bank data might not be consistent with the annually reported data by each country’s Ministry of Health. Another limitation of this study is the World Bank data has only predictors at the highest level (national); therefore, the findings from this study might not be helpful for decisions at the individual and community levels. Despite these limitations, we anticipate that the analysis will provide meaningful and impactful results due to the large time span of time series data available for analysis. With 16 years of data available for 46 African countries, this study is the first of its kind to examine the relationship between external health expenditure and infant mortality in addition to public and private sources of health expenditure in the region.

## Conclusion

This study determined the impact of health care expenditure on infant mortality in SSA countries. The results provided evidence that total health care expenditure per capita was associated with the reduction of infant and neonatal mortality rates. The results also showed that both public and external sources of health care expenditure were significantly negatively associated with infant mortality.

Health expenditure is an important issue, which can dominate policy decisions at both national and international levels. Health expenditure is growing faster than GDP in most SSA countries. Increasing governments’ health care financing over the next years will be crucial in reducing mortality and improving health outcomes in the region. Therefore, there is a need for governments in the region to increase amounts allocated to health care service delivery. In the absence of financial support the viability of SDG achievement in Africa will be precarious. The findings from this study suggest that increased allocation of aid towards health purposes in the future could improve neonate and infant health outcomes.

In addition, the out-of-pocket health expenditure in the region should be decreased to reduce infant mortality and establish effective public-private partnerships to reduce infant and neonatal mortality. The high level of out-of-pocket spending or very low public spending in some low and middle-income countries stands out as one of the most troubling areas for public health policy. The finding from this study will add a current evidence to the scientific literature regarding health expenditure (public and external) and infant mortality in a similar nations of SSA.

## Supplementary information

**Additional file 1.** Supplementary material.

## Data Availability

The full dataset supporting the conclusions of this article is available upon request.

## Abbreviations

GDP: Gross Domestic Product
GHED: Global Health Expenditure Database
MDGs: Millennium Development Goals
OOP: Out Of Pocket
SDGs: Sustainable Development Goals
SSA: Sub-Saharan African countries
WDI: World Bank Development Indictors
WHO: World Health Organization
