# Supplementary material for: The effects of health expenditure on infant mortality in sub-Saharan Africa: evidence from panel data analysis
Source: Health Econ Rev. 2020 Mar 6;10:5. doi: 10.1186/s13561-020-00262-3 (PMC7060592; doi:10.1186/s13561-020-00262-3)
Supplement: Supplementary file 1 — Additional file 1. Supplementary material. [file 13561_2020_262_MOESM1_ESM.doc]

## *Supplementary information on measurement and definition of terms*

## [***Additional file 1. Supplementary material.***](https://static-content.springer.com/esm/art%3A10.1186%2Fs13561-019-0248-4/MediaObjects/13561_2019_248_MOESM1_ESM.pdf)

| Variables | Description |
| --- | --- |
| Health status (HS) | Represents child health outcome measures including, neonatal mortality and Infant mortality |
| Neonatal mortality rate  (NMR) | Number of deaths during the first 28 completed days of life per1000 live births in a given year |
| Infant mortality rate  (IMR) | The probability of a child death in a specific year or period dying before reaching the age of one |
| Total health expenditure  (THE) | Total health expenditure expressed as a percentage of gross domestic product (GDP). Covers spending on preventive and curative health services, family planning activities etc. |
| Public health expenditure  (PuHE) | Level of public spending on health expressed as percent of GDP. Includes spending from government budgets, external borrowing, grants and social health insurance funds |
| Private health  Expenditure (PrHE) | Level of private expenditure on health expressed as a percentage of GDP. Includes direct household (out-of-pocket) spending, private insurance, charitable donations and direct service payments by private corporations |
| External health expenditure (ExtHE) | Share of current health expenditures funded from external sources. External sources compose of direct foreign transfers and foreign transfers distributed by government encompassing all financial inflows into the national health system from outside the country. External sources either flow through the government scheme or are channelled through non-governmental organizations or other schemes. |
| Real GDP per capita  (RGDPpc) (Y) | Real GDP per capita measured in constant 2011 international dollars |
| Measles immunization | Percentage of children ages 12-23 months who received measles immunization before 12 months |
| Education (Educ) | Secondary school enrolment as percentage of gross school enrolment |
| Sanitation (S) | Percentage of population using an improved sanitation facility |
| HIV prevalence rate (HIV) | Estimated number of adults aged 15-49 years with HIV infection expressed as percent of total population in that age group |
| Urbanization (Urban) (U) | Percentage of population living in areas classified as urban according to the criteria used by each country |
| Population aged 14 years  and below (Pop1) | Population age group below or equal to 14 years expressed as percentage of total population |
| Population 65 years  and above (Pop2) | Population age group above 65 years expressed as percentage of total population |
